# T-2 Dedicated Burn Therapists and Verification Status: National Survey of U.S. Burn Centers

**DOI:** 10.1093/jbcr/irag033.002

**Published:** 2026-04-06

**Authors:** Audrey M O'Neil, Ingrid Parry, Derek O Murray, Rodica Ioana Muraru, Emma Holler, Soman Sen, Leigh J Spera

**Affiliations:** Richard M Fairbanks Burn Center, Indianapolis, IN; University of California Davis, Sacramento, CA; Diane & Bruce Halle Arizona Burn Center - Valleywise Health, Phoenix, AZ; Indiana University, Indianapolis, IN; Indiana University School of Medicine, Indianapolis, IN; University of California Davis Health, Sacramento, CA; Indiana University/Eskenazi Health, Indianapolis, IN

## Abstract

**Introduction:**

Burn survivors require ongoing therapy for years post-discharge to prevent contractures, restore independence, and manage scarring. Having trained and skilled occupational and physical therapists (OT/PT) in the follow-up outpatient (OP) burn clinic to address ongoing rehabilitation and reconstruction needs is essential for comprehensive long-term care. While the ABA verification process promotes OT/PT in OP burn clinics, barriers such as space, time, and staffing are common constraints for burn programs. Little is known about real-world integration of OT/PT within OP burn clinics. This study examined the utilization of OT/PT in OP burn clinics and examined associations with clinic structure, patient services, and verification status.

**Methods:**

U.S. burn centers were sent a 20-question electronic survey. Questions addressed clinic structure and resources, staffing utilization, and verification status. Responses were received from 54 (43%) of burn centers. Frequencies and percentages described categorical variables. Fisher’s exact and Chi-square analyses estimated associations using verification status and designated OP therapists as outcomes, respectively. Firth logistic regression was applied to account for small sample sizes.

**Results:**

Of the responding burn centers, 35 (65%) reported having dedicated OT/PT staff in the OP burn clinic, 33 (61%) were ABA-verified, and most (30, 56%) treated both adult and pediatric patients (Table 1). On bivariate analysis, the presence of designated OT/PT was significantly associated with verification status (*p*=.007). Centers with dedicated OT/PT were also more likely to place therapy orders directly in OP clinic (*p*<.001), provide therapy treatment at follow-up (*p*=.020) and after reconstruction surgery (*p*=.027), offer patient transportation for follow-up (*p*=.024), and have a compression garment program (*p*=.017). Verified centers were more likely to have burn surgeons responsible for reconstruction programs (85% vs. 15%), while in non-verified centers, responsibility was more evenly divided between burn surgeons (57%) and external plastic surgeons (43%). After controlling for burn reconstruction program, centers with designated OT/PT had over three times the odds of being verified compared to those without (OR = 3.5, *p*=.043).

**Conclusions:**

While ABA-verified burn centers were more likely to report dedicated OT/PT services in their outpatient clinics, it was the presence of these therapists that was associated with broader rehabilitation resources, including follow-up interventions and compression garment programs. These findings suggest that verification supports the integration of therapists, but that therapist presence itself is the critical factor driving comprehensive long-term rehabilitation.

**Applicability of Research to Practice:**

ABA verification promotes OT/PT integration, and therapist presence improves access and continuity of care.

**Funding for the study:**

N/A.

